# Thyroid autoimmunity in relation to islet autoantibodies in newly diagnosed type 1 diabetes mellitus in children and adolescents

**DOI:** 10.3389/fendo.2026.1822960

**Published:** 2026-05-07

**Authors:** Yingli Zhu, Hongxiu Yang, Conghui Hu, Lingyan Qiao, Cheng Li, Sicui Hu

**Affiliations:** Department of Endocrine Genetic Metabolism in Children, The Affiliated Hospital of Women and Children of Qingdao University, Qingdao, Shandong, China

**Keywords:** autoimmunity, children, islet autoantibodies, thyroid, type 1 diabetes mellitus

## Abstract

**Purpose:**

This study aimed to investigate the occurrence of thyroid autoantibodies at the onset of type 1 diabetes mellitus (T1DM) in children and adolescents, and to assess whether the presence of islet autoantibodies is associated with thyroid autoantibody presence.

**Methods:**

This retrospective cross-sectional study included 122 children and adolescents newly diagnosed with T1DM who visited Qingdao University Affiliated Women’s and Children’s Hospital between January 2022 and December 2024. We collected their blood glucose, glycated hemoglobin (HbA1c), C-peptide levels, and autoantibodies to islet cell antigen (ICA), glutamate decarboxylase (GADA), islet antigen-2 (IA-2A), insulin (IAA) and zinc transporter 8 (ZnT8A), thyroid peroxidase antibodies (TPOAb), and antithyroglobulin antibodies (TGAb).

**Results:**

84.4% of patients had at least one islet autoantibody, with IA-2A showing the highest positivity rate (64.8%) and ZnT8A the lowest (3.3%). No patient tested positive for all five antibodies. In comparison of 42 thyroid autoantibody (TA)-positive patients (34.4%) with 80 TA-negative patients, the TA-positive group was older (7.8 ± 3.0 years vs. 6.6 ± 3.1 years, P = 0.045). The GADA positivity rate (60.9% vs. 41.4%, P = 0.004) and IAA positivity rate (33.3% vs. 11.3%, P = 0.003) were significantly higher in the TA-positive group than in the TA-negative group, with a higher median GADA titer (29.3 U/ml vs. 5.2 U/ml, P = 0.029). Logistic regression analysis revealed that GADA positivity (OR = 3.18, 95% CI: 1.44-7.01, P = 0.004) and IAA positivity (OR = 3.94, 95% CI: 1.53–10.15, P = 0.004) were significantly associated with TA positivity. After adjusting for age, sex, BMI, and family history of diabetes, both remained independently associated with TA (aOR=2.68 and 4.06, respectively). Additionally, GADA titer showed a weak positive correlation with TPOAb titer (r = 0.204, P = 0.024).

**Conclusions:**

Positive GADA and IAA antibodies were independently associated with thyroid autoimmunity in children with newly diagnosed type 1 diabetes, and GADA titer correlated with TPOAb titer. These findings support the consideration of early screening and long-term monitoring of thyroid function in children with newly diagnosed type 1 diabetes, particularly those positive for GADA and/or IAA antibodies.

## 1 Introduction

Type 1 diabetes mellitus (T1DM) is a chronic metabolic disorder characterized by hyperglycemia due to insufficient insulin secretion. The majority of patients suffer from immune-mediated diabetes, caused by cellular mediated autoimmune destruction of pancreatic β-cells, usually leading to complete insulin deficiency (1). Globally, the incidence of type 1 diabetes is increasing, particularly among children and adolescents living in low-income and middle-income countries (2).

Type 1 diabetes is classified as either autoimmune (autoantibody positive [iAb+]) or idiopathic (autoantibody negative [iAb-]). The primary form of type 1 diabetes is iAb+, where islet autoantibodies (iAb) destroy pancreatic cellular infiltrate (2). Islet autoantibodies are commonly used in the diagnosis and prediction of type 1 diabetes mellitus. They are well established as the biomarkers of the underlying autoimmune pathogenesis. Glutamic Acid Decarboxylase (GADA), islet Cell Cytoplasmic (ICA), Insulinoma-Associated-2/Tyrosine Phosphatase (IA-2A), Insulin (IAA) and, Zinc Transporter-8 Autoantibodies (ZnT8A) are the most commonly used islet autoantibodies at diagnosis (3).

Individuals with T1DM exhibit an increased risk of other autoimmune disorders, which may influence their prognosis (4). The most common Associated Autoimmune Diseases (AADs) with measurable autoantibodies in patients with diabetes are thyroid disease, celiac disease, autoimmune gastritis and Addison’s disease (5). A common feature of these AADs is the presence of autoantibodies months or years before overt disease manifests. Detection of these autoantibodies at type 1 diabetes onset may prevent complications associated with delayed diagnosis of these disorders (6).

Among these, autoimmune thyroid disease (AITD) is the most common associated autoimmune disorder in T1DM (7). One notable association is the co-occurrence of AITD in 20%-30% of T1DM patients, and conversely, it has been reported that 4%-8% of AITD patients are complicated with T1DM (8). Additionally, the prevalence of thyroid autoantibodies and thyroid dysfunction is increased in subjects with non-thyroid autoimmune diseases such as T1DM (9). Therefore, screening for thyroid autoantibodies in T1DM may help in early detection of autoimmune thyroid disorders. Clinically, thyroid dysfunction is often associated with metabolic disturbances and may affect diabetes control. Hyperthyroidism may worsen glycemic control while hypothyroidism alters carbohydrate metabolism (9). Therefore, routinely screening in T1DM subjects allows early detection and treatment of thyroid dysfunction.

This study aimed to investigate the occurrence of thyroid autoantibodies at the onset of T1DM in children and adolescents, and to assess whether the presence of a specific islet autoantibody was associated with thyroid autoantibodies. The objective was to provide a basis for assessing thyroid function in children and adolescents with T1DM, and to minimize the risk of undiagnosed thyroid dysfunction.

## 2 Material and methods

### 2.1 Subjects

This was a retrospective cross-sectional study conducted at the Qingdao University Affiliated Women’s and Children’s Hospital in Qingdao, Shandong Province. We analyzed retrospectively the medical records of children and adolescents newly diagnosed with T1DM from January 2022 to December 2024. All patients met the criteria of American Diabetes Association for T1DM (10). All data were collected at a single time point (the time of T1DM diagnosis), and no longitudinal follow-up was performed, consistent with the cross-sectional design of this study. Patients were excluded if they lacked complete data for all five pancreatic autoantibodies (GADA, IAA, IA-2A, ZnT8A, ICA) or for both thyroid autoantibodies (TPOAb, TGAb). No imputation was performed. We reviewed the demographic data and the results of antibody tests. This study was approved by the Institutional Review Board of the hospital. The recommendations of the Declaration of Helsinki for biomedical research involving human subjects were followed.

### 2.2 Methods

Levels of blood glucose, glycosylated hemoglobin (HbA1c), insulin, C-peptide and all five pancreatic autoantibodies (IA-2A, ZnT8A, GADA, ICA, IAA) were evaluated within 24 hours of diagnosis, prior to insulin therapy initiation. GADA, IAA, and ICA were detected using chemiluminescence immunoassay on a Kaeser 6600 platform (Guangzhou Kangrun Biotechnology Co., Ltd., Guangzhou, China). Positivity was defined as GADA≥10 IU/mL, IAA≥20 IU/mL, and ICA≥20 IU/mL according to the manufacturer’s cutoff values. According to the manufacturer’s specifications, the intra-assay coefficient of variation (CV) was ≤ 8.0%, and the inter-assay CV was ≤ 15.0%. IA-2A was measured by radiobinding assay (KingMed Diagnostics, Guangzhou, China), with positivity defined as≥2 U/mL. According to the manufacturer’s specifications, the intra-assay and inter-assay CVs were within acceptable ranges. ZnT8A was detected by immunoblotting (KingMed Diagnostics, Guangzhou, China), with results interpreted as positive or negative, and quantitative titers were not available for this autoantibody. Screening for TA was performed using measurements of thyroid peroxidase antibody (TPOAb) and thyroglobulin antibody (TGAb). TA was defined as the presence of at least one thyroid autoantibody (11). TPOAb (normal range, 0–60 IU/mL) and TGAb (normal range, 0-4.5 IU/mL) were measured using direct chemiluminescence immunoassay on a Centaur XP platform (Siemens Healthcare Diagnostics). Calibration for all autoantibodies was performed according to the manufacturer’s instructions using standard calibrators provided with each kit.

### 2.3 Statistical analysis

Data are expressed as mean ± standard deviation, median (interquartile range) or proportion (%). Antibody titers were analyzed without transformation. Normality of continuous variables was assessed using the Shapiro–Wilk test. The independent sample t-test was used for normally distributed continuous variables, and the Mann–Whitney U test was used for non-normally distributed continuous variables. The chi-square test was used to compare percentages among different patient subgroups. Logistic regression analysis was used to assess the strength and independence of the associations. Non-parametric Spearman’s rank correlations were conducted to evaluate the correlation between antibody titers. Outcomes included pancreatic antibody positivity and TA. All statistical analyses were performed with IBM SPSS ver. 27.0. A P-value <0.05 was considered significant. This study was exploratory in nature, and no *a priori* sample size calculation was performed. The sample size was determined by the availability of complete autoantibody data.

## 3 Results

Of the 171 patients initially selected for the study, 49 were excluded because they lacked complete data for all five pancreatic autoantibodies and both thyroid autoantibodies. Thus, 122 patients were included in the final analysis. To assess whether exclusion due to incomplete autoantibody data introduced selection bias, we compared baseline characteristics between included and excluded patients (**Table 1**). No significant differences were observed in age (P = 0.418), sex (P = 0.981), or HbA1c (P = 0.342), suggesting that no significant differences were observed between included and excluded patients with respect to these baseline characteristics.

**Table 1:** Comparison of baseline characteristics between included and excluded patients.

Variable	Included(n=122)	Excluded(n=49)	P value
Age at diagnosis(yr)	7.0 (5.0-9.0)	7.0 (4.5-10)	0.418
Male sex (%)	60 (49.2%)	24 (49.0%)	0.981
HbA1c at diagnosis (%)	12.4 (10.6-13.9)	12.6 (11.5-14.4)	0.342

Data are presented as median (IQR) or n (%). P values were calculated using the Mann–Whitney U test for continuous variables and the chi-square test for categorical variables.

Of the 122 patients, 60 were boys (49.2%) and 62 (50.8%) were girls. The mean age at diagnosis of diabetes was 7.0 ± 3.1 years. Mean HbA1c at onset of diabetes was 12.3% ± 2.3% and the median (IQR) of the body mass index was 15.0 (13.8–16.2) kg/m². Most patients (103 of 122, 84.4%) had at least one pancreatic autoantibody at onset of T1DM, whereas only nineteen patients (15.6%) were negative for all five antibodies. In particular, the most frequent islet autoantibody was IA-2A (64.8%), followed by GADA (50.8%), ICA (33.6%), IAA (18.9%), and ZnT8A (3.3%) (**Table 2**). Among all patients, 28.7% were positive for a single antibody, 29.5% for two, 23.8% for three, and 2.5% for four. No patient was detected with all five antibodies.

**Table 2:** Clinical features of type 1 diabetes patients with and without thyroid antibodies.

Variable	Total (n=122)	TA positive (n=42)	TA negative (n=80)	P value
Sex (M:F)	60:62	16:26	44:36	0.076
Age at diagnosis (yr)	7.0 ± 3.1	7.8 ± 3.0	6.6 ± 3.1	0.045
BMI (Kg/m^2^)	15.0 (13.8-16.2)	14.8 (13.6-17.5)	15.0 (13.9-16.0)	0.808
FH of T1D + (%)	3 (2.5)	0	3 (3.8)	0.204
Insulin at diagnosis (μIU/ml)	1.7 (1.2-2.8)	1.9 (1.4-2.8)	1.6 (1.0-2.9)	0.380
C peptide at diagnosis (ng/ml)	0.4 (0.3-0.5)	0.4 (0.3-0.5)	0.4 (0.3-0.5)	0.953
HbA1C at diagnosis (%)	12.3 ± 2.3	12.7 ± 2.2	12.0 ± 2.4	0.101
islet Ab positive (%)	103 (84.4)	41 (97.6)	62 (77.5)	0.004
IA-2Ab (U/ml)	5.2 (0.5-15.6)	5.8 (0.5-17.2)	5.2 (0.4-14.7)	0.471
GAD (IU/ml)	8.4 (2.2-166.5)	29.3 (3.6-257.1)	5.2 (2.0-64.8)	0.029
IAA (IU/ml)	0.5 (0.2-3.3)	0.5 (0.2-2.5)	0.5 (0.3-3.6)	0.974
IA-2Ab Ab positive (%)	79 (64.8)	30 (71.4)	49 (61.3)	0.264
ZnT8 Ab positive (%)	4 (3.3)	0	4 (5.0)	0.141
GAD Ab positive (%)	62 (50.8)	29 (60.9)	33 (41.4)	0.004
IAA Ab positive (%)	23 (18.9)	14 (33.3)	9 (11.3)	0.003
ICA Ab positive (%)	41 (33.6)	17 (40.5)	24 (30.0)	0.244

Data are presented as mean ± standard deviation, or median with the interquartile range (IQR), or frequency (percentage). TA, Thyroid Autoantibody; BMI, Body mass index; FH of T1D +, Family history of type 1 diabetes in first-degree relatives.

42 patients (34.4%) were positive for thyroid autoantibodies at diagnosis of T1DM. Among the 42 patients with positive thyroid autoantibodies, only one did not have islet autoantibodies (97.6% vs. 77.5%, P = 0.004). Age represents a significant difference between positive and negative TA groups (7.8 ± 3.0 years vs. 6.6 ± 3.1 years, P = 0.045). No significant differences were observed in sex (P = 0.076), HbA1c (P = 0.101), BMI (P = 0.808), insulin level (P = 0.380), or C-peptide level (P = 0.953) between the two groups. Compared with TA-negative patients, those in the TA-positive group had higher rates of GADA positivity (60.9% vs. 41.4%, P = 0.004) and IAA positivity (33.3% vs. 11.3%, P = 0.003). The median GADA titer in the positive TA group was also significantly higher than that in the negative group (29.3 IU/ml vs. 5.2 IU/ml, P = 0.029). However, there were no significant differences in the positive rates of IA-2A (P = 0.264), ZnT8A (P = 0.141), and ICA (P = 0.244) antibodies between the two groups (**Table 2**).

In the univariate logistic regression analysis, both GAD antibody positivity (OR = 3.18, 95% CI: 1.44 - 7.01, P = 0.004) and IAA antibody positivity (OR = 3.94, 95% CI: 1.53 - 10.15, P = 0.004) were significantly associated with TA positivity. No significant associations were found for IA-2A (P = 0.265) or ICA (P = 0.246) antibodies. ZnT8A could not be analyzed due to the very low number of positive cases (**Table 3**).

**Table 3:** Univariate logistic regression analysis of factors associated with thyroid antibodies positivity.

Factor	OR	95%Cl	P value
IA-2Ab (Positive vs. Negative)	1.58	0.71-3.5	0.265
ZnT8 (Positive vs. Negative)	NA	NA	NA
**GAD (Positive vs. Negative) **	**3.18**	**1.44-7.01**	**0.004**
**IAA (Positive vs. Negative) **	**3.94**	**1.53-10.15**	**0.004**
ICA (Positive vs. Negative)	1.59	0.73-3.46	0.246

OR, Odds ratio; CI, Confidence interval.

Data are presented as OR (95% CI). ORs were derived from univariate binary logistic regression.

Bold values indicate statistical significance (P < 0.05).

These associations for GAD and IAA antibodies remained significant after adjustment for potential confounders including age, sex, BMI, and family history of diabetes, with adjusted ORs of 2.68 (95% CI: 1.11-6.44, P = 0.028) and 4.06 (95% CI: 1.42-11.62, P = 0.009), respectively (**Table 4**).

**Table 4:** Multivariate logistic regression model for independent factors associated with thyroid autoantibody positivity.

Independent variable	Model 1			Model 2			Model 3			Model 4		
	OR	95%Cl	P value	OR	95%Cl	P value	OR	95%Cl	P value	OR	95%Cl	P value
GAD positivity	2.59	1.14-5.89	0.023	2.79	1.69-6.67	0.021	2.82	1.18-6.76	0.020	2.68	1.11-6.44	0.028
IAA positivity	3.03	1.14-8.09	0.027	4.07	1.42-11.66	0.009	4.15	1.45-11.90	0.008	4.06	1.42-11.62	0.009

Model 1 was unadjusted. Model 2 was adjusted for age and sex; Model 3 was additionally adjusted for BMI on the basis of Model 2; Model 4 was further adjusted for family history of diabetes mellitus on the basis of Model 3. P < 0.05 was considered statistically significant; OR, odds ratio; CI, confidence interval.

Non-parametric Spearman’s rank correlations revealed a weak positive correlation between GAD titer and TPOAb titer (r = 0.204, P = 0.024), while no significant correlation was observed with TGAb titer (P = 0.652). IAA titer was not significantly correlated with TPOAb (P = 0.647) or TGAb (P = 0.575). Similarly, IA-2A titer showed no significant correlation with TPOAb (P = 0.533) or TGAb (P = 0.487) (**Table 5**).

**Table 5:** Correlations between islet autoantibody titers and thyroid autoantibody titers.

Islet Autoantibody	TPOAb titer		TGAb titer	
	r value	P value	r value	P value
GADA Titer	0.204	0.024	0.041	0.652
IAA Titer	-0.042	0.647	-0.051	0.575
IA-2A Titer	-0.057	0.533	-0.063	0.487

P < 0.05 was considered statistically significant.

## 4 Discussion

Identifying the risk of thyroid autoimmunity in children with T1DM at an early stage is important, because thyroid dysfunction is often associated with metabolic disorders, which often affect glycemic control of T1DM. Although numerous studies have demonstrated an increased risk of thyroid autoimmune diseases in individuals with T1DM, little research has examined the presence and association of islet autoantibodies and thyroid autoantibodies in children newly diagnosed with T1DM. Autoantibodies may also be correlated with specific clinical manifestation, disease severity, and rate of progression. This study explored the relationship between islet autoantibodies and thyroid autoantibodies in Chinese children newly diagnosed with T1DM, providing evidence for the screening and management of thyroid autoimmunity in pediatric T1DM patients in clinical practice.

In this study, children with positive thyroid antibodies were significantly older at diagnosis. The positivity of GADA and IAA was significantly associated with TA, and this association remained significant after adjusting for confounding factors such as age and sex. GAD antibody titer showed a weak positive correlation with TPOAb titer. These findings suggested that positivity for GAD and IAA antibodies is independently associated with thyroid autoimmunity and supported the consideration of enhanced screening and follow-up for thyroid autoimmunity in children with newly diagnosed T1DM who are positive for GAD and/or IAA.

The positive rate of TA in newly diagnosed T1DM children in this study was 34.4%, which was basically consistent with the positive rate of thyroid autoantibodies in T1DM patients reported in some previous studies (30%-40%) (8). This indicated that as a population with autoimmune diseases, T1DM patients have a higher likelihood of concurrent thyroid autoimmune abnormalities than the general population. Research indicated that more than 20% of patients with T1DM had thyroid autoantibodies and 50% of them progressed to clinical AITD. Conversely, in some patients initially presenting isolated AITD, symptoms of insulin-requiring diabetes appeared after a variable period of time (12). T1DM and AITD often clustered in individuals and families, reflecting a shared genetic susceptibility rather than organ-specific processes acting in isolation. This close relationship was largely explained by a shared genetic background, primarily attributed to specific HLA class II haplotypes. Among them, the HLA antigens DQ2 and DQ8, tightly linked with DR3 and DR4, were the major common genetic predispositions (13). In addition to HLA, non-HLA susceptibility genes such as CTLA-4 and PTPN22 had also been implicated in both diseases (13). Furthermore, Anaya et al. (14) found in their study that 25.5% of families of T1DM patients had at least one first-degree relative with an autoimmune disease, compared with only 9% in control families. And the most frequent AADs registered in first-degree relatives (FDR) of cases were AITD and T1DM. These results indicated that autoimmune diseases cluster within families of T1D patients. Beyond genetic susceptibility, immune cross-reactivity has been proposed as another mechanism linking islet and thyroid autoimmunity. A study (15) found that certain HLA molecules could present autoantigens from both the pancreas and thyroid, inducing thyroid and islet specific T-cell responses.

At the same time, we observed that the prevalence rates of GADA, IAA, and ICA in this study were 50.8%, 18.9%, and 33.6% respectively, which were lower than the results reported in other countries. Among them, studies in eastern India and South Korea indicated that the prevalence of type 1 diabetes GADA was as high as over 70%, while a study in northern Iran pointed out that ICA was the most common diabetes antibody in T1DM patients, with a prevalence rate of 66.2% (9, 16–18). A survey on type 1 diabetes in Henan Province, China, showed that the prevalence rates of GADA, ICA, and IAA were 28.1%, 22.8%, and 2.6% respectively, which were much lower than those in our study (19). Another study from China (20) indicated that the prevalence of GADA was 56.3% at the time of diagnosis, decreased to 50.5% one year later, and further dropped to 43.3% after 3–5 years. The reported prevalence of these autoantibodies in T1DM may vary depending on factors such as the duration of the disease, the age of onset, and the method of antibody detection. Genetic heterogeneity across populations, environmental factors and methodological differences may also contribute to these variations.

This study found that the diagnostic age of patients in the TA-positive group was significantly higher than that of the TA-negative group. This was consistent with the findings of Anca et al., which indicated that the prevalence of thyroid antibodies increases with age and the duration of diabetes (21). There was no significant difference in gender distribution between the two groups, which was inconsistent with the findings of most previous studies suggesting a higher incidence of autoimmune diseases in women (4, 21). This might be related to the relatively small sample size of this study. Additionally, our study included patients aged 3 to 13 years, who were in the prepubertal stage. During this period, the impact of sex hormones on immunity has not yet become pronounced (22), which may explain the absence of a gender difference in our cohort. Future research may expand the sample size and include adult patients for further validation.

This study confirmed that the presence of GAD antibodies in newly diagnosed children with T1DM was significantly correlated with thyroid autoimmunity, which was consistent with most previous studies (5, 9, 23–25). For instance, Versha et al. found in their study that among children with T1DM in Pakistan, those with positive anti-GAD antibodies had a higher incidence of thyroid diseases, and there was a significant correlation between positive GADA and TGAb (24). Regarding the relationship between antibody titers, our study only found a statistically significant but weak correlation between GADA titer and TPOAb titer, which explained only a very small proportion of the variation in TPOAb titer. Therefore, this finding should be interpreted cautiously, and its clinical relevance should not be overstated. A Swedish study also indicated that GADA and ZnT8A were associated with thyroid autoimmunity at the time of clinical diagnosis of T1DM (25).

In this study, the presence of IAA positivity was also independently associated with positive thyroid autoantibodies, which was consistent with the conclusion of Seok Jung et al. (11). However, there was no correlation in antibody titers. Some studies have indicated that the presence of IAA decreased with age (26). Therefore, this lack of correlation might be related to the age distribution differences in our study population. Furthermore, Anita et al. found in their study that the presence of GADA, particularly ZnT8 autoantibodies, was associated with AITD in non-obese adults with newly diagnosed diabetes (27). The discrepancy between our findings and those of Anita et al. (27) may be partly explained by the fact that the prevalence of ZnT8A in our pediatric cohort was extremely low (3.3%), which limited statistical power to detect any association. Further studies with larger sample sizes and broader age ranges are needed to clarify this relationship.

Diabetes-associated autoantibodies are serological markers of β-cell autoimmunity. These markers are essential diagnostic tools to distinguish between type 1, type 2, monogenic, and other forms of diabetes. The prevalence of islet autoantibodies varies in accordance with patients’ age and ethnic origin, as well as disease duration. In addition to having a diagnostic value in type 1 diabetes, periodic testing of autoantibodies can help identify people with an increased risk of developing the disease, particularly in children of parents with T1DM (17).

The prevalence of thyroid dysfunction increases with the presence of thyroid autoantibodies. Screening for anti-thyroid antibodies in patients with T1DM may help in the early detection of autoimmune thyroid diseases. For children with newly diagnosed T1DM who have positive GADA and IAA, it may be reasonable to consider screening for thyroid autoantibodies, particularly TPOAb, and repeating the test annually, as there is a possibility of antibody conversion to positive in the future (28). In case of positivity, additional work-up may be considered, including functional and structural examinations. If necessary, intervention measures may be considered (such as T4 or antithyroid, iron and vitamin B12 supplementation, gluten-free diet) (4).

Thyroid hormones can affect the metabolism of sugar, fat and protein, and thus may influence the control of diabetes. A study (29)investigated the relationship between thyroid hormone levels and glucose and lipid metabolism in children with primary T1DM and normal thyroid function. It found that TSH and FT3 were negatively correlated with HbA1c and suggested that thyroid hormone levels are closely related to glucose and lipid metabolism. Therefore, monitoring thyroid function in T1DM patients may facilitate early detection of abnormalities and could potentially improve the disorder of glucose and lipid metabolism and reduce the risk of diabetes-related complications. A study from Uganda also suggested evaluating the thyroid function of children and adolescents with T1DM to help reduce the risk of undiagnosed thyroid dysfunction (30).

This study has several limitations. First, this was a retrospective, single-center study in a specific region of China with a relatively modest sample size, which limits the generalizability of the findings to other populations or settings. Additionally, the low prevalence of ZnT8A positivity (only 4 patients) limited the reliability of statistical comparisons involving this antibody. Therefore, conclusions regarding ZnT8A should be considered preliminary. Second, our multivariate logistic regression model included six predictors based on 42 events, and the modest sample size may increase the risk of overfitting. Third, several potential confounders (pubertal status, symptom duration, family history of thyroid disease, iodine status) were not available in this retrospective study and could not be adjusted for. The absence of these variables may introduce residual confounding, and the findings should be interpreted with caution. Fourth, we did not assess thyroid function (e.g., TSH, FT4) and had no longitudinal follow-up. Therefore, we could not determine whether patients who were initially negative for TA would become positive over time, nor whether those with TA positivity would develop clinically significant thyroid dysfunction during follow-up. Finally, although we found no significant differences between included and excluded patients, selection bias cannot be completely excluded. Future multi-center, prospective studies with larger sample sizes and longitudinal follow-up are needed to validate our findings and to evaluate the progression of thyroid dysfunction in this population.

## 5 Conclusions

In summary, the prevalence of TA in newly diagnosed T1DM patients in this study was 34.4%. GADA and IAA positivity were associated with the presence of thyroid autoantibodies. For children with newly diagnosed T1DM, particularly those positive for GAD and/or IAA antibodies, it may be reasonable to consider enhancing screening for thyroid autoimmunity and follow-up of thyroid function, to help reduce the risk of undiagnosed subclinical thyroid dysfunction that could potentially affect diabetes control as well as growth and development.

## Data Availability

The original contributions presented in the study are included in the article/supplementary material. Further inquiries can be directed to the corresponding author.
